# COVID-19-induced acute kidney injury and chronic kidney disease: comment

**DOI:** 10.1590/2175-8239-JBN-2022-0115en

**Published:** 2022-09-05

**Authors:** Rujittika Mungmunpuntipantip, Viroj Wiwanitkit

**Affiliations:** 1 Independent researcher, Private Academic Consultant, Bangkok, Thailand.; 2 Joseph Ayo Babalola University, Ikeji-Arakeji, Nigeria.

Dear Editor,

We would like to comment on the article entitled “Differences between COVID-19-induced acute kidney injury and chronic kidney disease patients”^1^. According to Aroca-Martínez et al.^1^, AKI-NRF patients had considerably higher mortality rates, higher need for invasive ventilation, and higher D-dimer levels. We both agree that COVID-19 may result in kidney issues. At the same time, we should be aware that the patient’s renal condition may have been compromised before COVID-19, and that COVID-19 itself may have exacerbated the condition. The pre-COVID-19 data are not available in the current study, and clinical history alone is insufficient to exclude pre-COVID-19 issues. Additionally, it’s possible that the patient already has COVID-19, a disease that is both common and underdiagnosed^2^. To reach a definitive conclusion, a future study based on participants with known pre-COVID-19 health, immunological, and renal laboratory-confirmed data and a specific work-up to rule out previous asymptomatic COVID-19 may be helpful.
